# *Trichosporonaceae* as (Re-)Emerging Pathogens: A Warning to the Medical Community

**DOI:** 10.3390/jof12030167

**Published:** 2026-02-26

**Authors:** Yasmim Passos Lima, Ricardo Villela Bastos, Victor Quinet de Andrade Bastos, Lucas Quinet de Andrade Bastos, João Renato Hipólito, André Netto Bastos, Cláudio Galuppo Diniz, Vania Lucia Da Silva, Vanessa Cordeiro Dias

**Affiliations:** 1 Graduate Program in Biological Science, Federal University of Juiz de Fora—UFJF, Rua José Lourenço Kelmer, s/n, São Pedro, Juiz de Fora, MG 36036 900, Brazil; yasmimpassos14@gmail.com; 2 Cortes Villela Laboratory, Avenida Barão do Rio Branco, 2406/4 andar, Juiz de Fora, MG 36016 904, Brazil; rvillelabastos@yahoo.com.br (R.V.B.); victor@cortesvillela.com.br (V.Q.d.A.B.); lucas@cortesvillela.com.br (L.Q.d.A.B.); joao@cortesvillela.com.br (J.R.H.); 3 Department of Morphology, Federal University of Juiz de Fora—UFJF, Rua José Lourenço Kelmer, s/n, São Pedro, Juiz de Fora, MG 36036 900, Brazil; 4 Department of Parasitology, Microbiology, and Immunology, Federal University of Juiz de Fora—UFJF, Rua José Lourenço Kelmer, s/n, São Pedro, Juiz de Fora, MG 36036 900, Brazil; claudio.diniz@ufjf.br (C.G.D.); vania.silva@ufjf.br (V.L.D.S.)

**Keywords:** *Trichosporonaceae*, *Trichosporon asahii*, epidemiology, fungal infection, clinical outcomes, emerging pathogens

## Abstract

Background: The *Trichosporonaceae* family includes genera such as *Trichosporon*, *Apiotrichum*, and *Cutaneotrichosporon*, which are components of the human microbiota but may cause infections under conditions such as immunosuppression, prolonged hospitalization, invasive procedures, and broad-spectrum antimicrobial use. Objectives: This study aimed to describe the clinical and epidemiological characteristics of hospitalized and outpatient individuals with positive cultures for *Trichosporonaceae* species in Juiz de Fora, Minas Gerais, Brazil, and to correlate these findings with antifungal susceptibility profiles. Methods: Clinical isolates collected between 2020 and 2023 were identified using the Vitek 2^®^ system, and antifungal susceptibility was assessed by disk diffusion. Clinical and epidemiological data were obtained from electronic health records. Results: Among 40 isolates, *Trichosporon asahii* predominated (92.5%). Most cases involved hospitalized individuals (83.8%), mainly from intensive care units (81.8%). Respiratory infections and acute renal failure were the most common reasons for admission. The mean hospital stay was 34.8 days, and overall mortality reached 51.6%. Most individuals were male (77.5%) and older than 61 years (57.5%). Urine was the most frequent specimen (52.5%), and invasive infections predominated (87.5%). Corticosteroid use and invasive devices were common, and prior antibiotic use occurred in most cases. Only 35.0% of patients received antifungal therapy, predominantly fluconazole. Conclusions: *Trichosporonaceae* infections, particularly those caused by *T. asahii*, are associated with critically ill patients and high mortality, highlighting the need for early diagnosis, appropriate therapy, and continuous surveillance.

## 1. Introduction

The order Trichosporonales (phylum Basidiomycota, class Tremellomycetes) encompasses a diverse group of yeast-forming fungi, some of which have gained increasing recognition as opportunistic human pathogens of clinical relevance [1]. The *Trichosporonaceae* family consists of genera such as *Trichosporon*, *Apiotrichum*, *Cutaneotrichosporon*, *Effuseotrichosporon*, and *Haglerozyma*. Recent taxonomic re-evaluations have identified new genera. Various species have been shown to possess pathogenic potential and virulence factors, especially in clinical isolates [2].

Among the most frequently reported species within the genera comprising the *Trichosporonaceae* family are *Trichosporon* (including *Trichosporon asahii*, *Trichosporon inkin*, *Trichosporon ovoides*, *Trichosporon asteroides*, *Trichosporon coremiiforme*, *Trichosporon faecale*, *Trichosporon japonicum*, *Trichosporon lactis*, and *Trichosporon dohaense*), *Apiotrichum* (including *Apiotrichum mycotoxinivorans*, *Apiotrichum domesticum*, *Apiotrichum montevideense*, and *Apiotrichum loubieri)*, *Cutaneotrichosporon* (including *Cutaneotrichosporon dermatis*, *Cutaneotrichosporon cutaneum*, *Cutaneotrichosporon jirovecii* and *Cutaneotrichosporon mucoides*), *Effuseotrichosporon* (*Effuseotrichosporon vanderwaltii*) and *Haglerozyma (Haglerozyma chiarellii*) [2].

**Figure 1:**
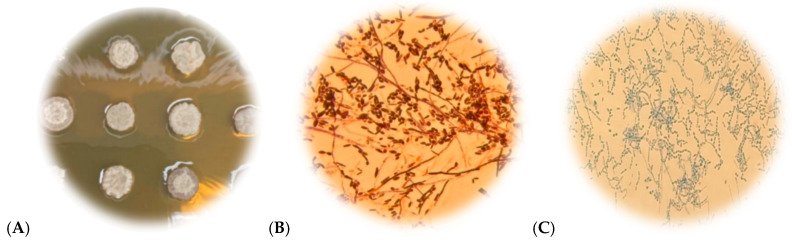
Macroscopic and microscopic features, respectively, were observed in isolates of species from the *Trichosporonaceae* family. Legend: (**A**) Macromorphology of *Trichosporon* spp. colonies on Sabouraud dextrose agar, showing dry, cerebriform colonies with white to cream coloration. (**B**) Micromorphology of Gram-stained *Trichosporon* spp., revealing pseudohyphae and arthroconidia. (**C**) Micromorphology of *Trichosporon* spp. stained with cotton blue, showing arthroconidia.

These fungi show characteristic macromorphology when grown on Sabouraud Dextrose Agar (SDA). They usually form dry, cerebriform colonies in white, beige, or cream colors [3,4]. Microscopically, they exhibit yeast-like structures, including blastoconidia, pseudohyphae, and arthroconidia. Arthroconidia are a distinctive trait of *Trichosporon* spp. [5,6] (Figure 1).

While morphological characteristics are useful, identifying *Trichosporon* species with conventional methods is often challenging and often yields inconclusive results. This difficulty is compounded by the absence of standardized in vitro susceptibility testing protocols [7]. Consequently, as observed with other fungal genera, molecular techniques are now recommended for the accurate identification of yeast species formerly classified under the genus *Trichosporon*. Taxonomic revisions have led to the reclassification of several clinically relevant species into three distinct genera: *Trichosporon*, *Apiotrichum*, and *Cutaneotrichosporon* [2].

These fungi are part of the healthy human microbiota and colonize the skin, nails, hair, and the respiratory and gastrointestinal tracts, as well as mucosal surfaces, albeit transiently. They are also present in a variety of environmental reservoirs, including soil, water, decomposing wood, and bird and bat droppings [8]. These yeasts can act as opportunistic pathogens in the presence of predisposing factors such as immunosuppression, prolonged hospitalization, invasive procedures, or the use of broad-spectrum antimicrobials [5,9].

Superficial infections caused by *Trichosporonaceae* such as white piedra, a benign, chronic condition characterized by irregular, light-colored nodules along the hair shaft composed of blastoconidia, pseudo-hyphae, and arthroconidia, are relatively common, especially in immunocompetent hosts. Other cutaneous manifestations include dermatitis and onychomycoses [5,10].

Invasive infection, though less frequent than superficial manifestations, is a significant clinical concern due to high morbidity and mortality. These infections mainly affect hospitalized or critically ill individuals. Those with hematologic malignancies, organ dysfunction, or in intensive care units (ICUs) are at higher risk [11,12]. The growing incidence is linked to an increasing number of immunocompromised patients. Many undergo chemotherapy, immunosuppressive therapies, broad-spectrum antibiotics, invasive procedures, or solid organ transplantation. These conditions increase susceptibility and lead to poor clinical outcomes [1,7].

These infections are associated with high morbidity and mortality, representing a considerable challenge due to the limited therapeutic arsenal [13]. Currently, voriconazole is recommended as the first-line treatment based on in vitro sensitivity data, which also highlight posaconazole as an effective option against clinical isolates of *Trichosporon* spp. [14,15]. More recently, isavuconazole has gained prominence as a valuable alternative to voriconazole, offering a comparable antifungal spectrum with fewer drug interactions, reduced adverse effects, and predictable pharmacokinetics [16,17]. Amphotericin B and fluconazole may also be used, often in combination therapy, depending on the clinical scenario [18]. Treatment duration typically extends for 14 days in superficial infections, with longer courses required for invasive cases, particularly in immunocompromised individuals due to the high mortality rates [15].

The epidemiological distribution of clinically relevant *Trichosporon* species was assessed in a Brazilian population comprising 112 individuals. The species most frequently isolated from normal perigenital skin was *C. cutaneum* (29.46%), followed by *T. inkin* (10.71%). *C. mucoides* (8.92%) and *T. asahii* (6.25%) were also detected. Skin colonization was more prevalent among individuals aged 21–30 years (48.2%) and 31–40 years (25.0%). In clinical samples from urine and catheters, *T. asahii* was the predominant species (76.5%; *n* = 23), followed by *T. inkin* (16.6%; *n* = 5). The highest frequency of isolates from these samples was observed in the 71–80 age group (36.7%), followed by individuals aged 61–70 years (26.7%) [2,19].

With the recognition of *Trichosporonaceae* as emerging pathogens, it is vital to understand their clinical relevance, distribution, and antifungal susceptibility. Therefore, this study aimed to describe the clinical and epidemiological characteristics of hospitalized and non-hospitalized individuals with cultures positive for *Trichosporonaceae* species in Juiz de Fora, Minas Gerais, Brazil, between 2020 and 2023, and to correlate these findings with the antifungal susceptibility profiles of the isolates. This approach may support early recognition of infection, guide therapeutic decisions, and enhance the surveillance of these microorganisms.

## 2. Material and Methods

This was an experimental, descriptive, and cross-sectional study involving 40 unique clinical isolates of *Trichosporonaceae*, collected from hospitalized or non-hospitalized individuals. The samples were obtained from a clinical microbiology laboratory located in Juiz de Fora, Minas Gerais, Brazil, over a period from January 2020 to December 2023.

The participating institution is a private hospital with an approximate capacity of 160 beds and includes various specialized units, such as neonatal and adult intensive care units, coronary and neurological wards, surgical and general medical departments, and outpatient services.

All procedures were performed in accordance with ethical guidelines, following approval from the Human Research Ethics Committee of the Federal University of Juiz de Fora (CAAE 18611019.6.0000.5147) and after obtaining informed consent from all participants.

### 2.1. Study Participants

The inclusion criteria encompassed individuals with laboratory-confirmed positive cultures for *Trichosporonaceae*, irrespective of demographic variables such as age, sex, or unit origin (hospitalized or non-hospitalized). To prevent duplication, repeated isolates from the same individual were excluded from the analysis.

### 2.2. Review of Medical Records

An analysis of electronic medical records was conducted for individuals with *Trichosporonaceae* isolates. The variables extracted included: age, sex, unit of origin (hospitalized or non-hospitalized), inpatient unit, length of hospital stay, reason for hospitalization, clinical specimen, previous use of antimicrobials, use of antifungals after the diagnosis, use of corticosteroids within the 30 days prior to fungal culture testing, use of invasive devices, risk factors, and clinical outcome. All information was recorded and organized in a digital spreadsheet for further analysis (Excel^®^ version 2023).

### 2.3. Fungal Culture

Clinical specimens were inoculated onto Sabouraud Dextrose agar (Neogen of Brazil, Lansing, MI, USA), according to recommendations [19], and incubated at 35 °C for up to 30 days. After, the fungal isolates were preserved in sterile 2 mL tubes containing sterile distilled water, following the method outlined by Diogo et al. (2005) [20].

Morphological examinations and Gram staining were performed to evaluate the growth, viability, and purity of the isolates.

### 2.4. Yeast Identification

Yeast isolates were identified through biochemical and physiological analyses using the Vitek 2^®^ automated system (bioMérieux, Marcy-l’Étoile, France), as recommended by the manufacturer.

The reference strain *T. asahii* ATCC 90039 (American Type Culture Collection, Manassas, VA, USA) was used as quality control, yielding a 99.9% identification match, thus confirming the system’s reliability.

### 2.5. Susceptibility Testing to Antifungal Agents

The susceptibility profile of the *Trichosporonaceae* isolates was determined based on the guidelines established by the *Clinical and Laboratory Standards Institute* (CLSI) [21]. Antifungal susceptibility was evaluated using the disk diffusion method, with the following antifungal agents, fluconazole (25 μg), voriconazole (1 μg), amphotericin B (20 μg), and caspofungin (5 μg), all provided by Liofilchem Diagnostics (Roseto degli Abruzzi, Italy).

Each test was performed in duplicate, including controls for each antifungal. After incubation at 35 °C for 24 h, the diameter of the inhibition zones surrounding each disk was measured.

The interpretive criteria followed those proposed by Pfaller et al. (2010) [22] for fluconazole and voriconazole, Menezes et al. (2012) [23] for amphotericin B, and the manufacturer’s specifications (Liofilchem et al., 2021) [24] and CLSI [25] for caspofungin.

### 2.6. Statistical Analysis

Descriptive statistical analysis was performed, including percentages, absolute frequency, range, and mean values for participant age. Statistical analyses were performed using bivariate methods. Categorical variables were compared using Fisher’s exact test due to small sample size and expected frequencies below five. Continuous variables were compared using the Mann–Whitney U test after assessment of non-normal distribution. A *p*-value < 0.05 was considered statistically significant.

## 3. Results

**Figure 2:**
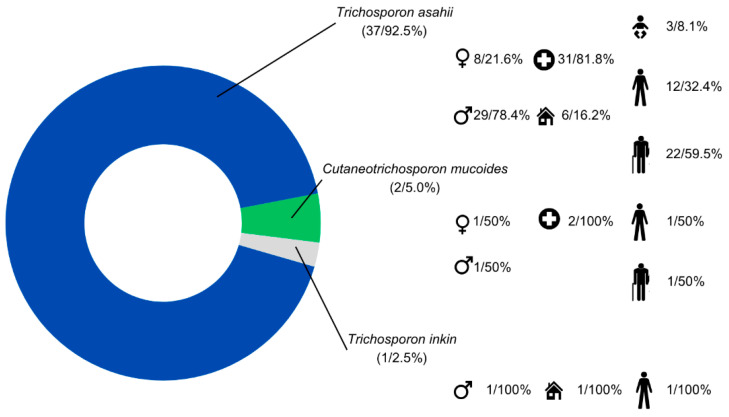
Distribution of *Trichosporon* and *Cutaneotrichosporon* species with corresponding clinical data. Legend: Icons represent sex (♂ male, ♀ female), hospitalization setting (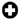 hospitalized or 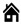 non-hospitalized), and clinical condition (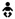 neonates, 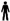 adults, and 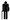 elderly (individuals older than 60 years)). The values indicate the absolute number and percentage of cases for each variable.

A total of 40 *Trichosporonaceae* isolates were recovered, predominantly from hospitalized individuals (*n* = 33/82.5%). Most cases occurred in male individuals (*n* = 31/77.5%) and individuals older than 61 years (*n* = 23/57.5%) (Figure 2).

Among the individuals infected by *T. asahii*, the most frequent species in this study, it mainly affected hospitalized elderly (*n* = 22/59.5%) and males (*n* = 29/78.9%). The two cases involving *C. mucoides* occurred in hospitalized male individuals: one adult and one elderly. *T. inkin* isolate was detected in a non-hospitalized adult male (Figure 2).

Defining invasive infection as that resulting from deep tissue penetration (e.g., urinary or respiratory tract) or hematogenous dissemination, most cases in this study met these criteria (*n* = 35/87.5%), with a mean hospital stay of 34.8 days (range: 1–102). Among individuals with available clinical data, most were admitted to intensive care units, particularly neurological ICUs (*n* = 11/33.3%). The most frequent causes of hospitalization were respiratory infection (*n* = 8/24.2%) and acute renal failure (*n* = 7/21.2%). Overall mortality reached 51.5% (*n* = 17). Prior corticosteroid exposure was reported in 63.6% (*n* = 21) of cases, and risk factors such as invasive procedures were observed in 37.5% (*n* = 15) of individuals (Table 1).

**Figure 3:**
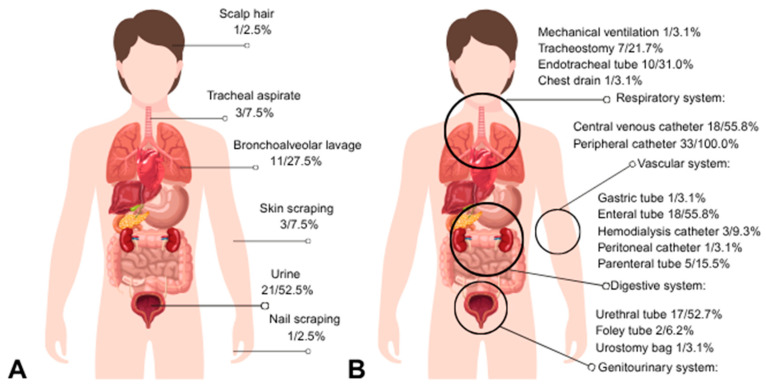
Sites of *Trichosporonaceae* isolation and frequency of invasive medical device use. Legend: (**A**) Anatomical distribution of clinical specimens from which *Trichosporonaceae* isolates were obtained (*n* = 40). (**B**) Distribution and frequency of use of invasive medical devices among individuals with *Trichosporonaceae* species (*n* = 33).

Urine was the most common clinical specimen (*n* = 21/52.5%), followed by bronchoalveolar lavage (*n* = 11/27.5%). All hospitalized individuals required invasive medical devices, and most individuals were exposed to multiple devices, reflecting the high clinical complexity of this study (Figure 3).

Prior antimicrobial exposure was observed in 72.5% (*n* = 29) of individuals, with combination therapy being the most common regimen (*n* = 24/60.0%). Following fungal diagnosis, only 35% (*n* = 14) of individuals received antifungal therapy, predominantly fluconazole (*n* = 7/17.5%). Notably, 65.0% (*n* = 26) of individuals did not receive any antifungal agents following diagnosis, underscoring potential gaps in targeted therapeutic management (Table 2).

To further evaluate potential clinical factors associated with unfavorable outcomes, a statistical analysis was performed comparing survivors and non-survivors among individuals with available clinical outcome data (*n* = 33). This analysis was conducted to identify variables potentially associated with mortality and is summarized in Table 3.

ICU admission was strongly associated with mortality, occurring in 88.2% of individuals who died compared to 18.7% of survivors (*p* < 0.001; OR 32.5; 95% CI 4.3–245.0). Prior antimicrobial exposure and previous corticosteroid therapy were also significantly associated with death (*p* = 0.018 and *p* = 0.004, respectively). Individuals who died were significantly older and had longer hospital stays (*p* = 0.004 and *p* = 0.040, respectively). Although antifungal therapy after diagnosis was more frequent among individuals who died, this association did not reach statistical significance (*p* = 0.080).

**Graph 1:**
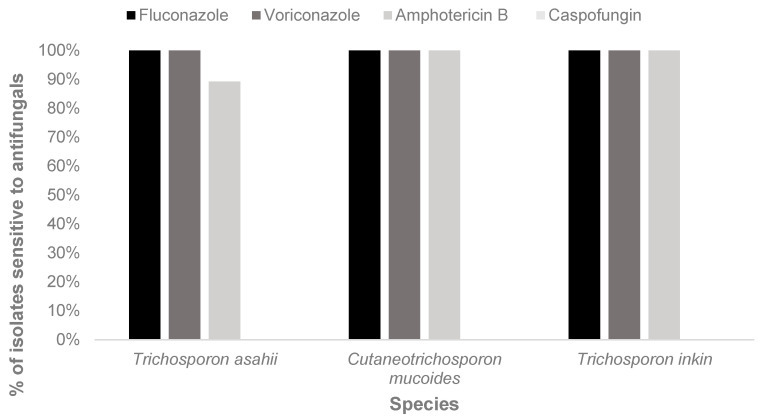
In vitro sensitivity of *Trichosporonaceae* isolates to antifungal agents.

All *C. mucoides* and *T. inkin* isolates were susceptible to fluconazole, voriconazole, and amphotericin B. Among *T. asahii* isolates, full susceptibility to azoles was observed, whereas resistance to amphotericin B was detected in four isolates (Graph 1).

## 4. Discussion

The present study provides clinical and epidemiological insights into *Trichosporonaceae* infections and identifies factors associated with mortality. *T. asahii* was the predominant species (92.5%), reinforcing its role as the most clinically relevant member of the genus, particularly in hospitalized and immunocompromised individuals. This predominance is consistent with previous studies describing *T. asahii* as the main etiological agent of invasive trichosporonosis [26,27]. Although less frequent, *C. mucoides* and *T. inkin* remain clinically relevant species and should not be overlooked, especially in specific clinical contexts [2,28].

The species identified in the present study are considered among the most clinically relevant in this genus [2,29]. The identification of *T. inkin* in a nail scraping specimen reinforces the role of this species in superficial infections, as described in studies associating it with white piedra, onychomycosis, and dermatomycoses [28,30,31]. These findings highlight the clinical diversity of *Trichosporonaceae* infections, which may range from superficial colonization to potentially fatal invasive disease.

The clinical profile observed in this study, characterized by a predominance of elderly male individuals, is consistent with previous reports of a higher susceptibility to opportunistic yeast infections in these populations. This distribution may be explained by the higher burden of comorbidities and immunosuppressive conditions commonly observed among elderly males, including malignancies, chronic renal disease, and diabetes mellitus, as well as age-related immune dysfunction (immunosenescence) [26,27]. In addition, sex-related immunological differences and increased exposure to invasive procedures, prolonged hospitalization, and indwelling medical devices in this group may further contribute to the increased risk of infection, reinforcing the role of host-related factors in the epidemiology of *Trichosporon* spp. infections [32,33].

Advanced age and longer hospital stay were also associated with mortality, reinforcing the role of host vulnerability and prolonged healthcare exposure in unfavorable outcomes.

The urinary tract was the main site of fungal isolation, particularly among hospitalized individuals undergoing urinary catheterization. This finding reinforces the urinary tract as an important reservoir for *Trichosporon* spp., especially in individuals with invasive devices [33,34,35]. Similar trends were found in a retrospective cohort from Mexico City (2019–2023), which identified 26 cases of *T. asahii* from urine cultures, mostly in male individuals (73%), all with urinary catheters, and the majority with central venous catheters (96%) and ICU admission (70%) [33]. Likewise, at King Abdulaziz University Hospital in Saudi Arabia, *T. asahii* accounted for 90.5% of *Trichosporon* isolates, with urinary tract infections predominating among elderly men [27].

In this study, we identified three individuals aged between birth and two years old. In early life, the gut mycobiome undergoes significant changes influenced by factors such as mode of delivery and exposure to intrapartum antibiotics. One study found that *Trichosporon* became the dominant fungal genus by 18 months of age, especially in vaginally delivered infants exposed to antibiotics during labor, where it accounted for up to 66% of the fungal community. These findings suggest that antibiotic exposure, although not directly affecting fungi, may alter fungal populations by altering the bacterial microbiota [36].

In the context of the human microbiota, this study’s results provide important insights into the relationship between fungal species and the presence of invasive devices, which can alter the host’s microbiological dynamics. The most frequent clinical sample in this study was urine, suggesting that fungal colonization of the urinary tract may be associated with invasive devices, such as peripheral catheters, central venous catheters, enteral tubes, and urinary catheters, which were the most commonly used in our study. Furthermore, the alteration of the microbiota caused by the presence of these devices may create an environment conducive to the proliferation of opportunistic fungi, which, by invading tissues or internal systems, can lead to severe infections, such as fungemia or urinary tract infections, especially in immunocompromised patients or those with predisposing conditions [37].

This risk is even more pronounced in ICUs, where individuals present greater immunological vulnerability and are exposed to various invasive medical devices [38]. The ability of fungi to survive for extended periods on inert surfaces, combined with the difficulty of removing them and the lack of effective, continuous disinfection practices, underscores the need for rigorous environmental surveillance and effective control strategies, particularly in critical areas such as ICUs [39,40].

The infectious picture is aggravated by the widespread ability of *Trichosporonaceae*, especially *Trichosporon* spp., to form biofilms, mainly in medical devices such as central venous catheters, urinary catheters, and cardiac implants [41,42]. These biofilms offer protection against antifungal agents and immune responses, promoting persistence and recurrence [5,43,44].

Prior antimicrobial exposure was also significantly associated with mortality. Broad-spectrum antimicrobial therapy may disrupt normal microbiota, promoting colonization and dissemination of opportunistic fungi. This finding reinforces the importance of antimicrobial stewardship and microbiological surveillance in hospital environments [36,37].

Prior corticosteroid use was reported in 63.6% of cases, highlighting its relevance as an important predisposing factor for opportunistic fungal infections [45]. In the present study, corticosteroid therapy was also significantly associated with mortality, reinforcing its clinical impact on disease outcomes. Corticosteroids impair both innate and adaptive immune responses by reducing neutrophil and macrophage function, inhibiting cytokine production, and suppressing T-cell-mediated immunity, which are essential mechanisms for controlling opportunistic yeasts [46]. Additionally, prolonged or high-dose corticosteroid therapy may disrupt mucocutaneous barriers and alter host microbiota, facilitating fungal colonization and subsequent invasion. In the context of *Trichosporon* spp. infections, prior corticosteroid exposure has been frequently associated with severe disease and unfavorable outcomes, particularly among elderly and immunocompromised patients, emphasizing the importance of careful risk assessment and early recognition of invasive fungal infections in this population [47,48].

Despite microbiological confirmation of infection, antifungal therapy was administered in only 35% of cases, revealing an important gap between laboratory diagnosis and clinical management. This discrepancy underscores a disconnect between laboratory diagnosis and clinical intervention. Considering the high mortality rate, one must question whether more assertive and timely use of antifungal agents could have improved patient survival. This finding serves as a critical warning and reinforces the urgent need for early therapeutic decisions aligned with microbiological findings in the management of invasive fungal infections.

Although antifungal therapy was more frequently administered to individuals who died, no statistically significant association with mortality was observed. This finding likely reflects confounding by indication, since antifungal treatment is more commonly initiated in patients with more severe clinical conditions. Additionally, the high proportion of patients who did not receive antifungal therapy after fungal isolation may reflect challenges in differentiating colonization from invasive infection and highlights potential gaps in therapeutic decision-making.

*T. asahii* has emerged as the most frequently reported pathogen among rare yeast infections in Latin America, accounting for 49.5% of the 495 cases reviewed across eight countries [49]. These infections are associated with severe clinical outcomes, including a crude mortality rate of 40.8%, which increases significantly in cases of fungemia. The affected population is predominantly male and spans a wide age range from neonates to the elderly, highlighting the opportunistic potential of this pathogen. In addition, prior surgical procedures and antibiotic use have been statistically linked to an increased incidence of *Trichosporon* infections, reinforcing the importance of the hospital environment as a key setting for disease acquisition [49].

These findings highlight the urgent need for enhanced diagnostic vigilance and effective therapeutic strategies, given the persistently high lethality of *T. asahii*, even in individuals treated with antifungal agents such as amphotericin B [23].

In this research, antifungal sensitivity was assessed using the disk diffusion method, focusing on key agents such as fluconazole, voriconazole, and amphotericin B. Despite its limited standardization by major regulatory bodies, this method is recognized for its simplicity, reproducibility in routine laboratory settings, and low cost. Although its use remains underreported in scientific studies, especially in studies of *Trichosporonaceae*, it offers a wide range of potential clinical applications.

Among treated individuals, azole derivatives were the most frequently prescribed antifungals. Although *Trichosporon* spp. generally demonstrates susceptibility to this class, therapeutic failures may occur due to biofilm formation, host-related factors, and pharmacological limitations of antifungal agents. In contrast, combination therapy, especially when associated with polyenes, showed better outcomes, as three of the four discharged individuals were on combined regimens with other antifungal classes, suggesting potential benefits of synergistic antifungal approaches.

Azole derivatives inhibit ergosterol biosynthesis at the plasma membrane, thereby altering membrane permeability and enzyme function [50]. Clinical isolates of *Trichosporon* are generally susceptible to azoles, but multi-resistant strains have already been reported [51].

Antifungal agents from the echinocandin class show limited activity against *Trichosporon* spp. because these fungi have low levels of 1,3-β-D-glucan in their cell walls, the primary target of echinocandins. As a result, echinocandins exhibit poor fungistatic or fungicidal effects against *Trichosporon*, making them ineffective in clinical practice [3,52]. For this reason, they are not recommended for the treatment of invasive trichosporonosis, and alternative antifungals such as azoles (e.g., voriconazole) should be used [3,52].

Polyene antifungals exert their effect by extracting ergosterol from fungal membranes, forming so-called “ergosterol sponges” that disrupt membrane integrity. These agents have both fungistatic and fungicidal activity [53]. However, studies have shown that *T. asahii* is not only intrinsically resistant to caspofungin but also exhibits greater resistance to amphotericin B than other *Trichosporon* species. Resistance to amphotericin B and caspofungin is particularly concerning, as these are among the most widely used systemic antifungals in clinical practice, especially as first-line agents in the treatment of yeast infections [54].

Laboratory antifungal susceptibility testing is crucial, as empirical use of commonly prescribed agents, such as echinocandins, may result in therapeutic failure. Antifungal selection should therefore be guided by microbiological and susceptibility data to improve treatment outcomes, reduce healthcare costs, and minimize mortality.

It is important to emphasize that isolation of *Trichosporon* spp., particularly from urine samples, does not necessarily indicate causality in fatal outcomes. Many individuals in this cohort presented severe underlying diseases and multiple risk factors for mortality. Due to the retrospective design and limited clinical data, attributable mortality could not be determined, and findings should therefore be interpreted as associations between fungal isolation and unfavorable outcomes rather than confirmation of causality.

Ongoing surveillance of antifungal sensitivity patterns is essential not only to guide effective therapy but also to prevent the emergence of resistant strains [10,55] and to support the development of new antifungal agents. The findings of this study reinforce the concern that delays in the clinical response to laboratory diagnoses may negatively influence outcomes, particularly among vulnerable patient populations [8,14,56].

On the other hand, although the increasing number of reported cases of infections caused by *Trichosporonaceae* species represents a significant clinical challenge, it also opens avenues for innovation. There is a clear need for ongoing research into new antifungal compounds and the improvement in existing ones, the refinement of laboratory diagnostic methods, and hospital protocols that prioritize early recognition and targeted therapy. Addressing these gaps is essential for reducing the mortality and morbidity associated with this emerging group of opportunistic pathogens. Finally, raising awareness about fungi is important [57,58].

This study provides real-world data on *Trichosporonaceae* infections, highlighting their occurrence in both community and hospital settings and their association with critically ill patients and high mortality. The integration of clinical, epidemiological, and antifungal susceptibility data strengthens the relevance of the findings and underscores important gaps in diagnosis and treatment, particularly the low rate of antifungal therapy after diagnosis. However, the small sample size, incomplete clinical data, and reliance on disk diffusion for antifungal susceptibility testing may limit the generalizability of the results. Despite these limitations, this study offers valuable insights into the clinical burden and therapeutic challenges posed by *Trichosporonaceae* infections, reinforcing the need for improved surveillance, early diagnosis, and timely antifungal management.

## 5. Conclusions

Infections caused by *Trichosporonaceae* represent a significant challenge in hospital settings due to their high lethality and frequent association with critically ill individuals. The findings of this study highlight the circulation of these fungi both in the community and within hospital environments, underscoring the need for enhanced clinical and laboratory surveillance. The high mortality rate observed among hospitalized individuals reinforces the severity of these infections. Although *Trichosporonaceae* isolates show good sensitivity to fluconazole and voriconazole, intrinsic resistance to caspofungin underscore the complexity of therapeutic management.

Additionally, the identification of a significant therapeutic gap, in which only 35% of individuals received antifungal treatment after diagnosis, reveals an important area for improvement in clinical care. The high proportion of individuals untreated with antifungals, combined with diagnostic delays and a lack of clinical suspicion, may have contributed to the unfavorable outcomes observed. These findings highlight the urgent need to raise awareness among healthcare professionals and implement standardized protocols for the timely initiation of appropriate antifungal therapy.

## Figures and Tables

**Table 1:** Clinical and epidemiological characteristics of individuals with *Trichosporonaceae* infections.

Clinical and Epidemiological Parameters	*Trichosporonaceae* (*n* = 40)
Type of infection: *n* = 40	
Surface	5 (12.5)
Invasive	35 (87.5)
Length of hospital stay (in days): average (range)	34.8 (1–102)
Origin non-hospitalized: *n* = 40	
Non-hospitalized Origin hospitalized: *n* = 33	7 (17.5)
Coronary unit	1 (3.1)
Impatient unit	9 (27.2)
Surgical center	6 (18.1)
General ICU	4 (12.2)
Neurological ICU	11 (33.3)
Neonatal ICU Reason for hospitalization: *n* = 33	2 (6.1)
Cancer	3 (9.1)
Hemorrhage	1 (3.1)
Hydrocele and spermatocele	1 (3.1)
Respiratory infection	8 (24.2)
Heart failure	1 (3.1)
Liver failure	1 (3.1)
Acute renal failure	7 (21.2)
Respiratory failure	2 (6.1)
Osteomyelitis	1 (3.1)
Septicemia	4 (12.2)
Pulmonary tuberculosis	1 (3.1)
Pressure ulcer	3 (9.1)
Clinical outcome: *n* = 33	
Hospital discharge	16 (48.5)
Death	17 (51.5)
Prior use of corticosteroids: *n* = 33	
No	12 (36.4)
Yes	21 (63.6)
Clinical condition: *n* = 40	
No	25 (62.5)
Yes	15 (37.5)
Yes—Hemodialysis	4 (10.0)
Yes—Prematurity/twins	1 (2.5)
Yes—Surgery	1 (2.5)
Yes—Transplant	2 (5.0)
Yes—Bronchoscopy	6 (15.0)
Yes—Bronchofibroscopy and video laparoscopy	1 (2.5)

**Table 2:** Prescription of antimicrobial drugs before and after the diagnosis of fungal infection in all individuals, according to electronic medical records.

Clinical and Epidemiological Parameters	*Trichosporonaceae* (*n* = 40)
Prior use of antimicrobials: 40	n (%)
No	11 (27.5)
Yes	29 (72.5)
Yes—Combination therapy	24 (60.0)
Yes—Ceftriaxone	1 (2.5)
Yes—Cefuroxime	1 (2.5)
Yes—Piperacillin-tazobactam	3 (7.5)
Use of antifungals after diagnosis: 33	
No	26 (65.0)
Yes	14 (35.0)
Yes—Combination therapy	3 (7.5)
Yes—Fluconazole	7 (17.5)
Yes—Itraconazole	3 (7.5)
Yes—Nystatin	1 (2.5)

**Table 3:** Factors associated with mortality in individuals with *Trichosporonaceae* infection (*n* = 33).

Variable	Survivors *n* (%)	Death *n* (%)	OR (95% CI)	*p*-Value
ICU admission	3 (18.7)	15 (88.2)	32.5 (4.3–245.0)	<0.001
Prior use of antimicrobials	11 (68.7)	17 (100)	16.3 (0.8–331.0)	0.018
Prior use of corticosteroids	6 (37.5)	15 (88.2)	11.5 (2.0–66.0)	0.004
Use of antifungals after diagnosis	4 (25.0)	10 (58.8)	4.3 (0.9–20.0)	0.080
Age *	Lower median	Higher median	—	0.004
Length of hospital stay *	Shorter median	Longer median	—	0.040

**Legend:** Data is presented as number (percentage) unless otherwise specified. Odds ratios (ORs) and 95% confidence intervals (CIs) were calculated using bivariate analysis. Categorical variables were compared using Fisher’s exact test. Continuous variables (*) were compared using the Mann–Whitney U test. A *p*-value < 0.05 was considered statistically significant.

## Data Availability

All data generated or analyzed during this study on *Trichosporon* spp. are included in this published article.
